# Effects of Fisetin, a Plant-Derived Flavonoid, on Response to Oxidative Stress, Aging, and Age-Related Diseases in *Caenorhabditis elegans*

**DOI:** 10.3390/ph15121528

**Published:** 2022-12-08

**Authors:** Suhyeon Park, Bo-Kyoung Kim, Sang-Kyu Park

**Affiliations:** 1 Department of Medical Sciences, General Graduate School, Soonchunhyang University, Asan 31538, Chungnam, Republic of Korea; 2 Department of Medical Biotechnology, Soonchunhyang University, Asan 31538, Chungnam, Republic of Korea

**Keywords:** fisetin, oxidative stress, lifespan, age-related diseases, DAF-16, autophagy

## Abstract

Fisetin (3,3′,4′,7-tetrahydroxyflavone), a flavonoid abundant in various fruits and vegetables, including apple, strawberry, and onion, shows several beneficial effects such as anti-oxidant, anti-inflammatory, and anti-tumor effects. The free radical theory of aging suggests that age-related accumulation of oxidative damage is the major cause of aging and that decreasing cellular oxidative stress can regulate aging. Here, we investigated the effects of dietary supplementation with fisetin on the stress response, aging, and age-related diseases. Fisetin reduced the cellular ROS levels and increased the resistance to oxidative stress. However, the response to UV irradiation was not affected by fisetin. Both the mean and maximum lifespans were significantly extended by fisetin; lifespan extension by fisetin was accompanied by reduced fertility as a trade-off. Age-related decline in motility was also delayed by supplementation with fisetin. Amyloid beta-induced toxicity was markedly decreased by fisetin, which required DAF-16 and SKN-1. Reduced motility induced by a high-glucose diet was completely recovered by supplementation with fisetin, which was dependent on SKN-1. Using a Parkinson’s disease model, we showed that degeneration of dopaminergic neurons was significantly inhibited by treatment with fisetin. Genetic analysis revealed that lifespan extension by fisetin was mediated by DAF-16-induced stress response and autophagy. These findings support the free radical theory of aging and suggest that fisetin can be a strong candidate for use in novel anti-aging anti-oxidant nutraceuticals.

## 1. Introduction

Reactive oxygen species (ROS) including superoxide anion (O_2_^−^), hydrogen peroxide (H_2_O_2_), and hydroxyl radical (OH∙) are generated as byproducts of aerobic metabolism. The major site of cellular ROS production is the mitochondria through its electron transport chain reaction [1]. Due to their inherent reactivity, ROS can cause oxidative damage in cells [1]. Cells have evolved an anti-oxidant defense system to eliminate harmful ROS. The enzymatic defense system is composed of anti-oxidant enzymes, such as catalase (CAT), superoxide dismutase (SOD), and glutathione peroxidase (GPx). ROS can also be scavenged by anti-oxidant molecules, such as vitamin E, vitamin C, and glutathione. However, excess ROS can overcome the cellular anti-oxidant defense system and cause oxidative damage to cellular macromolecules including DNA, proteins, and lipids. The accumulation of oxidative damage can lead to the impairment of pivotal functions within cells. Such accumulation has been suggested to be associated with the pathophysiology of diverse diseases and aging of an organism [1,2]. The free radical theory of aging suggests that ROS-induced oxidative damage is a major cause of aging [3]. Tissues in aged animals exhibit higher levels of cellular ROS and oxidative damages compared to those in young animals. The rate of ROS generation is inversely correlated with species’ maximum lifespan in mammals [4]. However, genetically manipulating anti-oxidant enzymes has failed to show consistent results to support the free radical theory of aging. Genetic knockout of CAT or SOD can reduce the resistance to oxidative stress and lifespan. However, over-expression of these genes could not induce lifespan extension in *Caenorhabditis elegans* [5]. Transgenic mice expressing extra copies of anti-oxidant genes including CAT, SOD, and GPx did not show a longevity phenotype, while knocking out of these genes showed decreased lifespans [6]. Interestingly, simultaneous over-expression of CAT and SOD significantly increased the lifespan of *Drosophila melanogaster* only for short-lived strains [7].

Accumulation of ROS due to age-related functional decline in mitochondria is more pronounced in energy-consuming tissues such as the skeletal muscles, heart, and brain [8]. The age-related increase in ROS plays an essential role in the apoptosis of myocytes in the aged heart and development of cardiovascular diseases including atherosclerosis [9]. Emerging evidence suggests that ROS-induced oxidative damage contributes to brain aging. Neuronal and glial cell death is an important cause of brain aging. It is promoted by high levels of ROS in the aged brain [10]. Elevated ROS is also correlated with an age-related increase in neuroinflammation, which can lead to neurodegeneration [10]. Previous studies have suggested that ROS is one of the primary causative factors of many age-related diseases including cancers and neurodegenerative diseases. ROS-induced oxidative damages in DNA, such as mutations, strand breakage, and cross-linking, are involved in cancer initiation and development [11]. Survival of cancer cells is enhanced by ROS through MAPK/ERT1/2, JNK, and PI3K/Akt signaling pathways [12]. In addition, ROS can activate tumor metastasis by inducing the transition of epithelial cells to mesenchymal cells and expression of matrix metalloproteinases [13]. Mutations in gene-encoding SOD can cause approximately 10% of familial amyotrophic lateral sclerosis (ALS), the most common type of motor neuron disease. Increased ROS-induced oxidative damage in cellular macromolecules has been reported in ALS patients [14].

Based on the free radical theory of aging, people have searched for bioactive compounds that can retard age-related changes, extend the lifespan, and prevent age-related diseases. The utmost attention has been given to phytochemicals, secondary metabolites produced by various plants for the protection against environmental stresses, infections, and pollutants. Bioactivities of phytochemicals include anti-oxidant, anti-inflammatory, and anti-aging activities [15]. Supplementation with several phytochemicals is also beneficial for age-related diseases, such as cancer, cardiovascular diseases, and neurodegenerative diseases [15]. Direct ROS scavenging activities have been found in kaempherol and luteolin [16]. Dietary intake of epicatechin or curcumin can increase SOD activity [17,18]. Resveratrol, a phytochemical found in red wine, has shown lifespan-extending effects in yeast, nematodes, and fruit flies. It also shows protective effects against cancer, Alzheimer’s disease (AD), and diabetes mellitus (DM) [19,20,21,22]. Dietary supplementation with quercetin, a flavonoid abundant in grapes, blueberries, and broccoli, showed a strong ROS scavenging activity and increased the lifespan of *C. elegans* [23]. Phytochemicals extracted from *Dioscorea alata L.* were shown to be able to extend the lifespan and reduce the accumulation of α-synuclein in a Parkinson’s disease (PD) model [24].

Fisetin (3,3′,4′,7-tetrahydroxyflavone) is a phytochemical synthesized in various fruits and vegetables and belongs to flavonoids. Flavonoids are composed of a fifteen-carbon skeleton with two benzene rings connected by a pyrane ring [25]. Among various bioactivities of flavonoids, the best described is their anti-oxidant activity, which is dependent on the number and configuration of hydroxyl groups [26]. Like other flavonoids, fisetin shows anti-oxidant, anti-viral, anti-bacterial, and anti-inflammatory activities [25]. A recent study revealed that fisetin demonstrates an anti-diabetic effect through its α-glucosidase inhibitor activity [27]. Fisetin can significantly reduce age-related accumulation of lipid peroxidation and protein oxidation [28]. In rats, cellular ROS levels are decreased, while the expression of anti-oxidant enzymes CAT and SOD is increased by fisetin [28]. The anti-carcinogenic activity of fisetin has also been reported through in vitro and in vivo studies. The growth of human lung cancer cells is inhibited by treatment with fisetin [29]. Fisetin can repress the proliferation and migration of human oral squamous cell carcinoma and inhibit tumor growth [30]. In aged tissues, fisetin can induce apoptosis specifically in senescent cells and reduce the level of cellular oxidative damage [31,32]. Fisetin was the most potent inhibitor for cellular senescence among ten flavonoids, including rutin, quercetin, myricetin, and so on [33]. Both of the flavonoids fisetin and kaempferol increased the resistance to oxidative stress, but only kaempferol reduced the accumulation of lipofuscin in *C. elegans* [34]. Dietary supplementation with fisetin in old mice can significantly increase both the mean and maximum lifespan [33]. Fisetin also shows beneficial effects on neurodegenerative diseases via its neuroprotective activity. Administration of fisetin can restore cognitive and memory impairments caused by D-galactose [35].

In the present study, we aimed to investigate anti-oxidant and anti-aging activities of fisetin and determine protective effects against age-related diseases using *C. elegans* as a model system. We also intended to identify underlying cellular mechanisms involved in bioactivities of fisetin. Survival after oxidative stress or ultraviolet (UV) irradiation was compared between untreated control and fisetin-treated groups. The effects of fisetin on lifespan, fertility, and locomotive activity were determined. Using genetic or nutritional models of age-related diseases, we studied the preventive effects of fisetin on AD, DM, and PD. Underlying mechanisms involved in lifespan extension were identified using genetic mutants and genetic knockout of age-related genes.

## 2. Results

### 2.1. Fisetin Shows Radical-Scavenging Activity and Reduces Cellular ROS Levels

**Figure 1:**
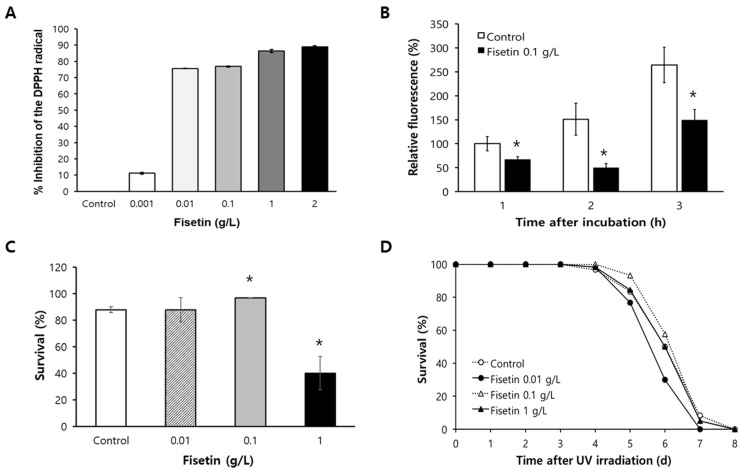
Effects of fisetin on oxidative stress and UV irradiation. (**A**) Radical-scavenging activities were measured with different concentrations of fisetin in vitro. (**B**) Cellular ROS levels in 7-day-old worms were compared between untreated control and fisetin-treated group. (**C**) Age-synchronized worms (*n* = 30) pre-treated with different concentrations of fisetin were incubated with H_2_O_2_ to induce oxidative stress for 6 h. Survival of worms was then compared to that of untreated control. (**D**) Effect of fisetin on response to UV irradiation was measured by recording daily survival rate after UV irradiation (*n* = 60). Error bar indicates standard error. *, significantly different compared to untreated control (*p* < 0.05). DPPH, 2,2-diphenyl-1-picrylhydrazyl; UV, ultraviolet.

To determine the radical-scavenging activity, the percent inhibition of DPPH radical was measured with different concentrations of fisetin. There was a dose-dependent increase in the radical-scavenging activity of fisetin (Figure 1A). The percent inhibition of DPPH radical was only 11.1 ± 0.52% for 0.001 g/L of fisetin. However, the inhibition effect was increased to 76.9 ± 0.37% for 0.1 g/L of fisetin and 89.0 ± 0.57% for 2 g/L of fisetin. In addition to in vitro radical-scavenging activity, we examined the effect of fisetin on cellular ROS levels in vivo. ROS levels were significantly decreased in fisetin-treated animals than in the untreated control. At all time-points after incubation, the fluorescence intensity resulting from cellular ROS was reduced significantly by dietary supplementation with fisetin. After 1 h of incubation, the percentage of relative fluorescence was 66.9 ± 6.02% in fisetin-treated worms, which was significantly decreased compared to that in the untreated control (100 ± 14.8%, *p* = 0.048). The percentages of relative fluorescence of the untreated control were 151.1 ± 33.3% and 264.2 ± 37.0% after 2 h and 3 h of incubation, respectively. Supplementation with fisetin decreased the percentage of relative fluorescence to 49.5 ± 8.98% (*p* = 0.006) and 149.1 ± 22.4% (*p* = 0.013) after 2 h and 3 h of incubation, respectively (Figure 1B).

### 2.2. Fisetin Increases Resistance to Oxidative Stress

Having observed radical-scavenging activity and reduced cellular ROS, we asked whether fisetin could affect resistance to oxidative stress. The survival of worms under oxidative stress was significantly increased after supplementation with 0.1 g/L of fisetin (Figure 1C). The percent survival after the induction of oxidative stress was not changed with 0.01 g/L of fisetin compared to that in the untreated control: 87.8 ± 2.22% in the untreated control and 87.8 ± 8.89% in worms treated with 0.01 g/L of fisetin. However, a significant increase in percent survival was observed with 0.1 g/L of fisetin (96.7 ± 0.00%, *p* = 0.016). Interestingly, a higher concentration of fisetin (1 g/L) decreased the survival rate under oxidative stress (40.0 ± 12.62%, *p* = 0.020). The response to another environmental stressor, UV irradiation, was not affected by dietary supplementation with fisetin (Figure 1D). Survival curves of untreated control and fisetin-treated worms were not significantly different. Based on these results of the response to oxidative stress, we decided to use 0.1 g/L of fisetin, which is 349.36 μM of fisetin, for the following experiments.

### 2.3. Fisetin Extends Lifespan of C. elegans and Reduces Fertility

**Figure 2:**
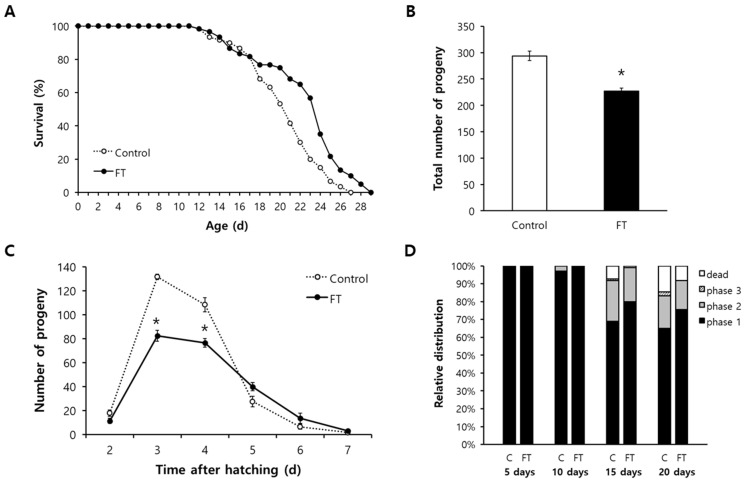
Effects of fisetin on lifespan, fertility, and age-related decline in motility. (**A**) Sixty age-synchronized worms were treated with or without fisetin throughout the whole lifespan. Survival curves of untreated control and fisetin-treated group were compared using the log-rank test. (**B**) Total number of progenies produced during a gravid period was counted (*n* = 12). (**C**) Time-course distribution of progeny number was monitored from 2 d after hatching until no progeny was produced. (**D**) Locomotive activity of each worm was classified according to response to mechanical stimuli: phase 1, a spontaneous locomotion without stimuli; phase 2, a whole-body locomotion only with stimuli; and phase 3, a head-only movement with stimuli. Error bar indicates standard error. C, untreated control; FT, 0.1 g/L of fisetin; *, significantly different compared to untreated control (*p* < 0.05).

To determine the effect of supplementation with fisetin on aging, we first compared the lifespan of worms treated with or without 0.1 g/L of fisetin. Both the mean and maximum lifespans were significantly increased by dietary supplementation with fisetin. The mean lifespan was increased from 20.4 d for untreated control to 22.4 d for fisetin-treated animals (*p* = 0.001). The maximum lifespans were 27 and 29 d for untreated control and fisetin-treated animals, respectively (Figure 2A). Independent repeated experiments also showed a significant increase in lifespan by fisetin (Table S1). The disposable soma theory suggests that there is an allocation of limited cellular resources during the aging process and that many lifespan-extending interventions can reduce reproduction as a trade-off for longevity. A fisetin-induced long lifespan was also accompanied by reduced fertility. The average total number of progenies produced during a gravid period was 293.8 ± 9.14 in the untreated control. However, the number was decreased to 227.3 ± 5.55 after supplementation with fisetin (*p* < 0.001) (Figure 2B). The time-course distribution of progeny numbers throughout the reproductive period also showed significant decreases in the number of progenies produced in the fisetin-treated group compared to that of the untreated control. Three-day-old untreated worms produced 131.7 ± 2.27 progenies, while 82.5 ± 4.72 progenies were produced by fisetin-treated worms at the same age (*p* < 0.001). The number of progenies hatched from 4-day-old mothers was reduced from 108.5 ± 5.90 in the untreated control to 76.5 ± 3.52 in the fisetin-treated group (*p* < 0.001) (Figure 2C).

### 2.4. Age-Related Decline of Locomotive Activity Is Delayed by Fisetin

Next, we examined the effect of fisetin on muscle aging characterized as sarcopenia, atrophy, and reduced motility [36]. The locomotive activity of each worm was classified into three phases as previously mentioned. The relative percent distribution of each phase was determined from young to old animals. As shown in Figure 2D, the locomotive activity reduced with aging. However, supplementation with fisetin retarded such age-related change in locomotive activity. There was no significant difference in locomotive activity between the untreated control and fisetin-treated group for young, 5- and 10-day-old, animals. However, the percentage of worms showing spontaneous locomotive activity without any stimuli, phase 1, was higher in the fisetin-treated group of old worms than that in the untreated control. In 15-day-old worms, 68.9% of worms showed phase 1 in the untreated control, while 80.0% of worms were classified as phase 1 in the fisetin-treated group. The percentage of worms categorized as phase 1 also increased in 20-day-old animals after supplementation with fisetin: 64.9% in the untreated control and 75.4% the in fisetin-treated group (Figure 2D).

### 2.5. Fisetin Delays Paralysis Induced by Aβ, Which Requires DAF-16 and SKN-1

**Figure 3:**
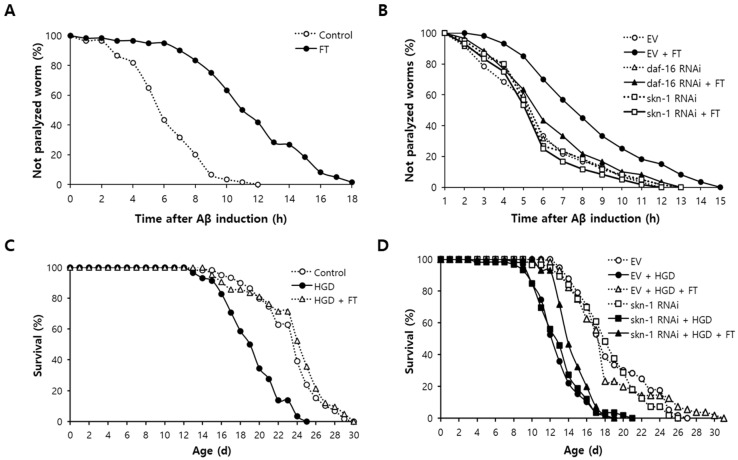
Amelioration of Aβ- and HGD-induced toxicity by fisetin. (**A**) The percentage of paralyzed worms by Aβ-induced toxicity was monitored every hour after Aβ induction in muscle tissues (*n* = 60). (**B**) Effect of daf-16 or skn-1 knockdown on inhibitory activity of fisetin against Aβ-induced paralysis was measured using RNAi. (**C**) Survival curves of animals were compared among untreated control, HGD-treated group, and HGD and fistein-treated group to evaluate the effect of fisetin on DM (*n* = 60). (**D**) Role of fisetin on HGD-induced toxicity was examined in worms fed with EV or skn-1 RNAi clone. Statistical analysis of survival curves was performed usig the log-rank test. Aβ, amyloid beta; FT, 0.1 g/L of fisetin; EV, empty vector; RNAi, RNA interference; HGD, high-glucose diet.

The effect of fisetin on AD was determined using a genetic disease model of AD. CL4176 strain expresses human Aβ transgene in muscles, which can lead to paralysis in *C. elegans* [37]. Dietary supplementation with fisetin significantly delayed paralysis caused by Aβ induction. To quantitatively determine the effect of fisetin on Aβ-induced paralysis, we compared the time in which 50% of worms were paralyzed between untreated and fisetin-treated groups. In the untreated control, the time in which 50% of worms were paralyzed was 6.3 h after the induction of Aβ transgene. However, it was extended up to 11.7 h for worms pre-treated with fisetin (*p* < 0.001) (Figure 3A). Genetic knockdown of stress-regulating transcription factors *daf-16* and *skn-1* completely abolished the inhibitory effect of fisetin on paralysis. There was a significant increase in time when 50% of worms were paralyzed by fisetin in control treated with empty vector (EV): from 4.9 h to 7.5 h (52.9% increase, *p* < 0.001). On the contrary, fisetin failed to delay Aβ-induced paralysis in worms in which the expression of *daf-16* was blocked. The time needed for 50% of worms to be paralyzed was 5.2 h for the untreated control and 5.6 h for the fisetin-treated group (*p* = 0.448). Knockdown of *skn-1* also eliminated the positive impact of fisetin on Aβ-induced toxicity. The time in which 50% of worms were paralyzed was not significantly different between the untreated control and fisetin-treated group (5.1 h and 4.7 h, respectively, *p* = 0.426) (Figure 3B). Independent replicative experiments showed similar results. There was a significant preventive effect of fisetin on toxicity in EV control. However, there was no change in Aβ-induced paralysis of worms whose expression of *daf-16* or *skn-1* was blocked (Table S2).

### 2.6. Reduced Survival by High-Glucose Diet (HGD) Is Recovered by Fisetin through SKN-1

HGD is widely used as a nutritional model of DM in *C. elegans* [38]. As shown in Figure 3C, worms fed with HGD exhibited significantly increased mortality. The mean lifespan was 23.4 d for untreated control and 19.4 d for worms treated with HGD (*p* < 0.001). The decreased survival due to HGD was markedly recovered by dietary supplementation with fisetin. The mean lifespan of worms treated with HGD and fisetin simultaneously was 23.8 d, which was a significant increase compared to that of HGD-only-treated group (*p* < 0.001) (Figure 3C). A previous study has revealed that SKN-1 is involved in HGD-induced toxicity in *C. elegans* [39]. The preventive effect of fisetin on HGD-induced toxicity disappeared with genetic knockdown of *skn-1*. In EV-treated control, the mean lifespan was decreased from 18.8 d without any supplementation to 12.9 d with HGD (*p* < 0.001). Simultaneous treatment with HGD and FT recovered the mean lifespan up to 18.3 d (*p* < 0.001 in comparison with HGD only). However, the decreased mean lifespan by HGD did not revert to normal after treatment with fisetin when the expression of *skn-1* was repressed. The mean lifespans were 18.3 d, 13.1 d, and 14.6 d in the untreated control, HGD-treated group, and both HGD and fisetin-treated group, respectively (Figure 3D). The results obtained with repeated experiments are shown in Table S3.

### 2.7. Fisetin Prevents Degeneration of Dopaminergic Neurons

**Figure 4:**
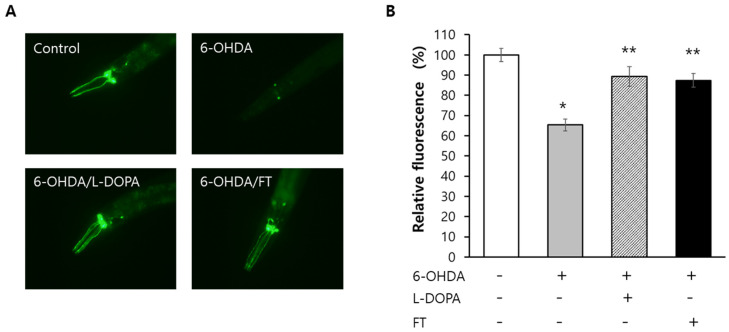
Recovery of degeneration in dopaminergic neurons by fisetin. (**A**) Expression of GFP in dopaminergic neurons was shown in untreated control. Experimental groups were treated with different combinations of 6-OHDA, L-DOPA, and fisetin. (**B**) Fluorescence observed in each group was quantified using Image J program and relative percent fluorescence was compared to untreated control. Error bar indicates standard error. 6-OHDA, 6-hydroxydopamine; L-DOPA, L-3,4-dihydroxyphenylalanine; FT, 0.1 g/L of fisetin; *, significantly different compared to untreated control (*p* < 0.05); **, significantly different compared to 6-OHDA-treated group (*p* < 0.05).

To examine the effect of fisetin on PD, a BZ555 strain expressing GFP specifically in dopaminergic neurons was employed. Degeneration of dopaminergic neurons was observed in worms treated with 6-hydroxydopamine (6-OHDA). L-3,4-dihydroxyphenylalanie (L-DOPA) restored dopaminergic neurons as expected. Surprisingly, supplementation with fisetin also showed a preventive effect on dopaminergic degeneration. The effect of fisetin was similar to that of L-DOPA (Figure 4A). Quantification of fluorescence revealed that the relative percent fluorescence was decreased from 100.0 ± 3.27% in the untreated control to 65.4 ± 2.96% in the group treated by 6-OHDA (*p* < 0.001). It was restored to 89.4 ± 4.91% when L-DOPA and 6-OHDA were used in treatment simultaneously (*p* < 0.001 compared with 6-OHDA-treated group). The relative percent fluorescence was 87.4 ± 3.36% in worms treated with both 6-OHDA and fisetin, which was significantly different from that in 6-OHDA-treated group (*p* < 0.001) but not significantly different from that in L-DOPA-treated group (*p* = 0.736) (Figure 4B). All three repeated experiments showed reproducible results for effects of 6-OHDA, L-DOPA, and fisetin on the degeneration of dopaminergic neurons (Table S4).

### 2.8. DAF-16-Induced Response and Autophagy Are Involved in Lifespan Extension by Fisetin

**Figure 5:**
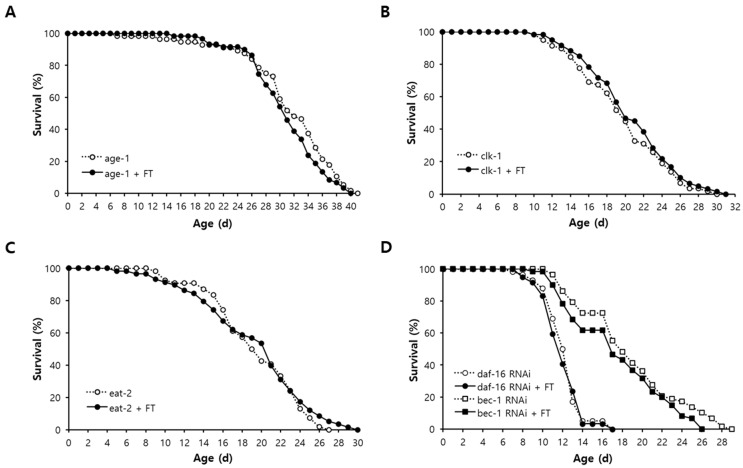
Effect of fisetin on lifespan of long-lived mutants. Effects of supplementation with fisetin on long lifespans of *age-1* (**A**), *clk-1* (**B**), and *eat-2* (**C**) were examined with sixty age-synchronized worms. (**D**) Survival curves of worms with or without fisetin were compared when the expression of *daf-16* or *bec-1* was repressed. Genetic knockdown of *daf-16* and *bec-1* was performed using RNAi clones. The log-rank test was employed for statistical analysis of survival curves. FT, 0.1 g/L of fisetin; RNAi, RNA interference.

**Figure 6:**
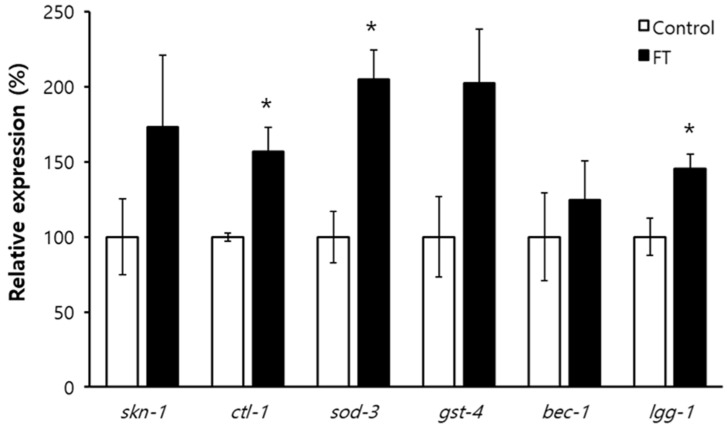
Quantitative RT-PCR of genes modulating anti-oxidant response and autophagy. Total RNA was extracted from 9-day-old worms and used for quantitative RT-PCR. Relative percent expression of each gene in fisetin-treated worms was calculated comparing to the expression of the same gene in untreated control (100%). Error bar indicates standard error. *, *p* < 0.05 compared with control; FT, 0.1 g/L of fisetin.

In order to elucidate the underlying mechanisms involved in fisetin-induced longevity, the effect of fisetin on lifespan of long-lived mutants was examined. The long lifespan of *age-1* due to reduced insulin/IGF-1-like signaling was not affected by supplementation with fisetin (Figure 5A). There was no additional lifespan extension by fisetin in *clk-1* mutants in which lifespan was extended by decreased mitochondrial electron transport chain reaction with less production of ROS as a result (Figure 5B). Dietary supplementation with fisetin failed to further increase the lifespan of *eat-2*, a genetic model of dietary restriction (DR) (Figure 5C). These findings indicate that lifespan-extending mechanisms of fisetin overlap with all three cellular pathways known to confer longevity phenotype in *C. elegans.* They suggest that common longevity-assuring mechanisms are involved in lifespan extension by fisetin. We determined the role of the anti-oxidant response and autophagy in fisetin-induced longevity. Knockdown of *daf-16*, a FOXO transcription factor regulating the expression of anti-oxidant genes, abolished the effect of fisetin on the lifespan. No significant change in lifespan by fisetin was detected when the expression of *daf-16* was inhibited. The mean lifespans were 12.3 d and 12.0 d in the untreated control and fisetin-treated group, respectively (*p* = 0.746). The lifespan-extending effect of fisetin also disappeared when the expression of *bec-1*, one of the major autophagic genes in *C. elegans*, was repressed. The mean lifespans of untreated control (18.8 d) and fisetin-treated group (17.5 d) were not statistically different in worms fed with *bec-1* RNAi clone (*p* = 0.155) (Figure 5D and Table S5). The expression levels of downstream targets of DAF-16, *ctl-1*, *sod-3*, and *gst-4* were induced in worms supplemented with fisetin. Compared to the untreated control, worms supplemented with fisetin showed a 1.6-fold increase in the expression of *ctl-1* (*p* = 0.025), a 2.0-fold increase in *sod-3* (*p* = 0.016), and a 2.0-fold increase in *gst-4* (*p* = 0.084). Although the mRNA level of *bec-1* was not altered by fisetin, the expression of another autophagic gene, *lgg-1*, was significantly increased by the supplementation with fisetin, showing a 1.5-fold increase in the fisetin-treated group compared to that in the untreated control (*p* = 0.043) (Figure 6).

## 3. Discussion

The free radical theory of aging suggests that increased ROS in aged tissues is one of the major causal factors of aging. Interventions that can reduce cellular ROS might retard the aging process. Dietary supplementation with anti-oxidants such as resveratrol and vitamin E conferred a longevity phenotype in model organisms [19,21,22,40]. DR is the most promising nutritional intervention showing anti-aging and lifespan-extending activities [41]; DR reduced cellular oxidative damages caused by ROS [41]. In the present study, we showed that fisetin could work as a strong anti-oxidant both in vitro and in vivo by reducing cellular ROS levels. Almost every flavonoid, including fisetin, quercetin, and kaempferol, has anti-oxidant activity. The number and position of hydroxyl groups in flavonoids affects anti-oxidant activity [26]. Determination of anti-oxidant activity with standard oxidizer revealed the anti-oxidant hierarchy: morin > kaempferol/quercetin > fisetin > apigenin > luteolin > catechin > chrysin [42]. Supplementation with fisetin also significantly increased both the mean and maximum lifespans of *C. elegans*, supporting the free radical theory of aging. The longer lifespan caused by fisetin was accompanied by reduced fertility as a possible trade-off for longevity. Previous studies have also reported that long-lived animals treated with resveratrol exhibit reduced fertility [43]. The long-lived *age-1* mutants produced reduced numbers of progenies [44]. These findings can be explained by the disposable soma theory of aging, which hypothesizes that there should be a recompense of limited cellular resources for lifespan extension. Age-related decline in motility was delayed in animals supplemented with fisetin. Increased ROS generation has been proposed as a main driver of sarcopenia, a loss of muscle mass with aging [45]. The contractile function of skeletal muscle was also modulated by ROS [45]. Taken together, our findings suggest that fisetin can confer anti-oxidant and anti-aging effects in *C. elegans*, possibly by reducing cellular ROS.

Oxidative damage caused by ROS is believed to be one of the major causes of age-related diseases. Elevated oxidative damage and a decreased anti-oxidant defense system were observed in AD patients [10]. Decreased glutathione and increased oxidative damage in DNA, proteins, and lipids have been observed in the substantia nigra of patients with PD [46,47]. We observed a significant reduction in paralysis induced by human Aβ transgene in the AD model and a complete recovery of reduced survival by HGD in the DM model by supplementation with fisetin. Preventive effects of fisetin on the degeneration of dopaminergic neurons were also shown in our PD model. Previous studies have identified beneficial effects of fisetin on neuro-degenerative diseases. In an AD model, fisetin reduced the formation of Aβ plaque and tau-mediated neurofibrillary tangles and enhanced memory [48,49,50]. Fisetin decreased α-synuclein aggregation and inhibited dopaminergic neurodegeneration in a mouse model of PD [51]. A recent study has revealed that phlorizin, an anti-oxidant phytochemical distributed in many plants, can inhibit Aβ- and HGD-induced toxicity and prevent dopaminergic degeneration in *C. elegans* [52]. DAF-16 and SKN-1 are transcription factors that can modulate the response to oxidative stress. FOXO3, a human homologue of DAF-16, mediates oxidative stress, energy metabolism, and autophagy [53]. Genetic variation in FOXO3 is associated with the longevity phenotype in diverse human populations [54]. NRF-2, a mammalian homologue of SKN-1, regulates the response to oxidative stress and shows protective roles in cancer and neurodegenerative diseases [53]. DAF-16 and SKN-1 are required for preventive effects of phytochemicals on age-related diseases. Delayed paralysis by Aβ observed in phlorizin-treated animals is dependent on both DAF-16 and SKN-1 [52]. However, the inhibition of Aβ aggregation by otophylloside B requires only DAF-16, while reduced Aβ-induced toxicity by rose essential oil is mediated only by SKN-1 [55,56]. The protective effect of fisetin in the AD model shown in this study also required both DAF-16 and SKN-1. SKN-1 was identified as an intracellular regulator that mediates decreased survival under HGD [39]. We found that the inhibition of *skn-1* expression abolished the recovery effect of fisetin on HGD-induced toxicity. These findings suggest that fisetin can confer beneficial effects on age-related diseases possibly by modulating the response to oxidative stress. Despite various pharmacological activities, fisetin has not been used for therapeutic applications due to its low aqueous solubility (10.45 μg/mL) and bioavailability (44%) [25]. However, recent attempts to enhance the solubility and bioavailability using polymeric nanoparticles showed promising results in breast cancer cells and a PD model [57,58]. Recently, a clinical trial for the effect of dietary supplementation with fisetin on frail elderly syndrome was conducted in older postmenopausal women by Mayo Clinic, ClinicalTrials.gov Identifier: NCT03675724. On the market, dietary supplements containing 8 to 500 mg of fisetin per capsule are advertised for anti-aging and anti-inflammatory effects.

Several cellular mechanisms conferring the longevity phenotype have been identified in *C. elegans*. Reduced insulin/IGF-1-like signaling by mutations in *daf-2* or *age-1* can increase the lifespan via DAF-16 [59]. Mutations in genes involved in mitochondrial electron transport chain reaction, such as *clk-1* and *isp-1*, can extend the lifespan due to decreased ROS production [60,61]. Congenital dysfunction in pharyngeal pumping can lead to the DR condition and prolong the lifespan as a result [62]. Interestingly, the longevity effect of fisetin overlapped with that of all three mutants tested, *age-1*, *clk-1*, and *eat-2*. These findings indicate that fisetin acts on common downstream pathways involved in all three mechanisms. Previous studies have found that transcriptional activation of DAF-16 downstream targets and activated autophagy are overlapping central pathways involved in many lifespan-extending mechanisms [63,64]. Complete disappearance of lifespan extension by fisetin with *daf-16* RNAi and significant inductions of downstream targets of DAF-16 by fisetin suggest that DAF-16-induced transcriptional response is one of the underlying mechanisms associated with the lifespan extension by fisetin. Fisetin failed to increase the lifespan when the expression of *bec-1* was repressed. In addition, the expression of *lgg-1*, another autophagic gene, was up-regulated by supplementation with fisetin. This supports our hypothesis that fisetin can extend the lifespan of *C. elegans* via the activation of autophagy.

## 4. Materials and Methods

### 4.1. Worm Strains and Culture

Wild-type N2 and all transgenic strains were purchased from *C. elegans* Genetics Center (CGC, Minneapolis, MN, USA). CL4176 (dvls27 [*myo-3/Aβ1-42/let UTR, rol-6*]) containing a muscle-specific human Aβ gene and BZ555 (egls1 [*dat-1p::*GFP]) expressing GFP in dopaminergic neurons, were employed as genetic disease models for AD and PD, respectively. Three long-lived mutants, *age-1* (*hx546*), *clk-1* (*e2519*), and *eat-2* (*ad465*), were used to determine lifespan-extending mechanisms. Worms were maintained on Nematode Growth Media (NGM) agar plates (25 mM NaCl, 2.5 mg/mL peptone, 50 mM KPO_4_, 5 μg/mL cholesterol, 1 mM CaCl_2_, 1 mM MgSO_4_, and 1.7% agar) spread with *Escherichia coli* OP50 at 20 °C.

### 4.2. Radical Scavenging Assay

Fisetin (Sigma Aldrich, St. Louis, MO, USA, PHL82542) was dissolved and diluted in 95% ethanol. Different concentrations of fisetin solution were added to freshly prepared 0.2 mM DPPH (2,2-diphenyl-1-picrylhydrazyl) ethanolic solution (1:1) into a 96-well plate. Mixtures were incubated at 37 °C for 30 min in the dark. After incubation, the reduction in DPPH free radical was measured by reading the absorbance at 517 nm (*A*) with a spectrophotometer. The DPPH scavenging activity was calculated using the following equation: Inhibition of DPPH radical (%) = [*A*_control_ − (*A*_sample_ − *A*_blank_)/*A*_control_] × 100.

### 4.3. ROS Measurement

Cellular ROS levels were measured for 7-day-old worms (*n* = 20). Individual worm was incubated in each well of a 96-well black plate containing 195 μL of PBST and 5 μL of H_2_DCF-DA (Sigma-Aldrich, St. Louis, MO, USA) for 3 h. The fluorescence intensity of each worm was then recorded using a fluorescence multi-reader (Infinite F200, Tecan, Grodig, Austria).

### 4.4. Responses to Environmental Stresses

Three-day-old age-synchronized worms were transferred to fresh NGM plates spread with 100 μL of each diluted fisetin solution and incubated at 20 °C for 24 h. To expose worms to oxidative stress, thirty worms were transferred to a 96-well plate containing 1 mM hydrogen peroxide (H_2_O_2_) in S-basal medium without cholesterol (5.85 g sodium chloride, 1 g potassium phosphate dibasic, and 6 g potassium phosphate monobasic in 1 L sterilized distilled-water) individually. After 6 h, the survival of each worm was monitored. Sixty age-synchronized worms pre-treated with fisetin for 24 h were irradiated with 20 J/cm^2^/min of UV for 1 min using a UV crosslinker (BLX-254, VILBER Lourmat Co., Torcy, France). Live and dead worms were counted every day until all worms were dead.

### 4.5. Lifespan Assay

The lifespan was determined with sixty age-synchronized worms grown on NGM plates spread with OP50. 5-Fluoro-2′-deoxyruridine (12.5 mg/L) was added to inhibit internal hatching. The numbers of live and dead worms were recorded until all worms were dead. Live worms were transferred to fresh NGM plates daily during a gravid period and every two days after reproduction to prevent starvation. Lost, killed, or bagged worms during an assay were excluded from data.

### 4.6. Fertility Assay

Randomly selected 2-day-old mother worms were allowed to lay eggs on a fresh NGM plate for 24 h, with a single mother worm on a single NGM plate (*n* = 12). Then, each mother worm was transferred to a new NGM plate and the old plate containing eggs spawned from each mother worm was incubated at 20 °C for another 48 h. The number of progenies hatched from eggs during 48 h of incubation was counted. Mother worms were transferred to fresh NGM plates daily until no progeny was produced.

### 4.7. Measurement of Locomotive Activity

The locomotive activity of each animal was classified into three phases: phase 1, worms showing spontaneous locomotion without any stimuli; phase 2, worms moving once a mechanical stimulus was provided; phase 3, worms only moving their head in response to mechanical stimuli (*n* = 100). The relative distribution of worms classified at each phase was compared between untreated control and fisetin-treated worms at 5, 10, 15, and 20 days after hatching.

### 4.8. Paralysis Induced by Human Aβ Transgene

Eggs were laid from young adult CL4176 for 2 h at 15 °C. After 24 h, worms hatched from eggs were transferred to a 25 °C incubator and incubated for 24 h to induce the expression of human Aβ gene in muscle. After 8 h of induction, paralyzed worms (*n* = 60) were counted every hour.

### 4.9. Survival under HGD

HGD was employed as a nutritional disease model of DM. Age-synchronized worms were grown on NGM plates spread with 100 μL of 40 mM glucose (*n* = 60). As previously mentioned, in the lifespan assay, the numbers of live and dead worms were recorded every day until all worms were dead. Survival curves of untreated control and HGD-treated worms were compared using the log-rank test.

### 4.10. Degeneration of Dopaminergic Neurons

To degenerate dopaminergic neurons specifically, worms were transferred to NGM media containing 50 mM 6-OHDA and 10 mM ascorbic. After gentle mixing every 10 min for 1 h at 20 °C, worms were washed with M9 buffer three times and transferred to fresh NGM plates spread with OP50 and 12.5 mg/L of 5-Fluoro-2-deoxyruridine. Worms were then incubated at 20 °C for 72 h. Each worm was mounted on a slide glass padded with 2% agarose and 1 M sodium azide. The fluorescence microscope with 485 nm excitation filter and 530 nm emission filter was used to visualize the degeneration of dopaminergic neurons. The fluorescent intensity observed in the head region was quantified using Image-J software. L-DOPA was used as a positive control for the inhibition of dopaminergic degeneration.

### 4.11. RNAi with Bacterial Feeding

Bacterial RNAi clones for *daf-16*, *skn-1*, and *bec-1* genes were obtained from Ahringer RNAi library [65]. Double-stranded RNA was induced from each clone by adding isopropyl-β-D-thio-galactoside (IPTG, Sigma-Aldrich, St. Louis, MO, USA) to bacterial culture media. Cultured bacteria were used as a food source for worms from 3 days after hatching (*n* = 60). Empty vector (EV) clone was used as a negative control for RNAi.

### 4.12. Quantitative RT-PCR

Total RNAs were extracted from approximately 300 worms collected in M9 buffer 9 days after hatching using a Trizol reagent (Thermo Fisher Scientific, Waltham, MA, USA). A measure of 1 μg of total RNA was reverse-transcribed to cDNA for RT-PCR. Quantitative RT-PCR was performed using a ReverTra Ace qPCR RT Master Mix (TOYOBO), 2× SyGreen Mix Hi-ROX (qPCRBIO), and a StepOne Plus Real-Time PCR System (Applied Biosystems, Waltham, MA, USA). Sequences of primers used for each gene are shown in Table S6. The expression of *ama-1* was used for normalization and the relative expression level of each gene was calculated using the 2^−ΔΔCt^ method.

### 4.13. Statistical Analysis

Statistical analysis for survival after UV irradiation, lifespan, paralysis, and survival under HGD were performed using the log-rank test [66]. The log-rank test is a non-parametric Mantel–Cox test commonly used for a comparison between two survival curves. For the other experiments, independent *t*-test was performed using jamovi, open statistical software built on the R statistical language. A *p*-value lower than 0.05 was considered to be statistically significant. All experiments were repeated three times independently.

## 5. Conclusions

Fisetin, a flavonoid abundant in strawberries (160 μg/g wet food), apples (26.9 μg/g), onions (4.8 μg/g), persimmons (10.5 μg/g), and so on, showed strong anti-oxidant and anti-aging activities in *C. elegans*. The cellular reactive oxygen species levels and susceptibility to oxidative stress were significantly decreased by fisetin treatment. Supplementation with fisetin extended the lifespan accompanied by reduced fertility and delayed age-related decline in motility. In age-related disease models, fisetin showed beneficial effects on Alzheimer’s disease, diabetes mellitus, and Parkinson’s disease. Genetic analysis revealed that the lifespan-extending effect of fisetin was mediated by DAF-16-induced stress response and autophagy. These findings suggest that fisetin is a promising natural compound with potential use in nutraceuticals against aging and age-related diseases. Identification of other intracellular targets and effects on other age-related diseases are necessary to gain a better understanding of the anti-aging activity of fisetin. Further studies focusing on bioactivities and bioavailability in mammalian model systems should follow to be developed as an anti-aging nutraceutical.

## Data Availability

Data is contained within the article and Supplementary Material.

## Supplementary Materials

The following supporting information can be downloaded at: https://www.mdpi.com/article/10.3390/ph15121528/s1, Table S1: Primer set of each gene used for quantitative RT-PCR; Table S2: Effect of fisetin on lifespan of *C. elegans*; Table S3: Effects of fisetin and RNAi of *daf-16*/*skn-1* on Aβ-induced toxicity in *C. elegans*; Table S4: Effects of fisetin and *skn-1* RNAi on reduced lifespan by HGD; Table S5: Effect of fisetin on degeneration of dopaminergic neurons; Table S6: Effect of *daf-16* or *bec-1* knockdown on lifespan extension by fisetin.
