# Supplementary material for: Effects of Fisetin, a Plant-Derived Flavonoid, on Response to Oxidative Stress, Aging, and Age-Related Diseases in Caenorhabditis elegans
Source: Pharmaceuticals (Basel). 2022 Dec 8;15(12):1528. doi: 10.3390/ph15121528 (PMC9786162; doi:10.3390/ph15121528)
Supplement: Supplementary file 1 [file pharmaceuticals-15-01528-s001.zip › pharmaceuticals-2030220-supplementary.pdf]

Table S1. Effect of fisetin on lifespan of *C. elegans*.

	Fisetin (g/L)	Mean lifespan (d)	*P* value[1)]	% effect[2)]
1st experiment	0	20.4		
	0.1	22.4	0.001	9.8
2nd experiment	0	18.2		
	0.1	21.8	< 0.001	20.1
3rd experiment	0	20.2		
	0.1	23.0	< 0.001	13.8

[1)] *P* value was calculated using the log-rank test by comparing the survival of untreated control group (0 g/L fisetin) to that of fisetin-treated group (0.1 g/L fisetin).

[2)] % effects were calculated by (C-P)/C*100, where P is the mean lifespan of fisetin-treated group and C is the mean lifespan of untreated control group.

Table S2. Effects of fisetin and RNAi of *daf-16*/*skn-1* on Aβ-induced toxicity in *C. elegans*.

	Fisetin (g/L)	RNAi	Time when 50% of worms were paralyzed (h)	*P* value[1)]	% effect[2)]
1st experiment	0	EV	4.9		
	0.1	EV	7.5	< 0.001	52.9
	0	*daf-16*	5.2		
	0.1	*daf-16*	5.6	0.448	8.7
	0	*skn-1*	5.1		
	0.1	*skn-1*	4.7	0.426	-7.8
2nd experiment	0	EV	5.5		
	0.1	EV	6.9	0.013	25.5

	0	*daf-16*	5.4		
	0.1	*daf-16*	4.8	0.451	-11.1
	0	*skn-1*	6.5		
	0.1	*skn-1*	5.6	0.215	-13.3
3rd experiment	0	EV	4.9		
	0.1	EV	6.4	0.004	32.0
	0	*daf-16*	5.4		
	0.1	*daf-16*	4.9	0.263	-8.6
	0	*skn-1*	7.1		
	0.1	*skn-1*	7.4	0.453	3.7

1) *P* value was calculated using the log-rank test by comparing the rate of paralysis in untreated control group (0 g/L fisetin) to that in fisetin-treated group (0.1 g/L fisetin).

2) % effects were calculated by (*C*-*P*)/*C**100, where *P* is the mean lifespan of fisetin-treated group and *C* is the mean lifespan of untreated control group.

Table S3. Effects of fisetin and *skn-1* RNAi on reduced lifespan by HGD.

	Supplementation	RNAi	Mean lifespan (d)	*P* value
1st experiment		EV	18.8	
	HGD	EV	12.9	< 0.001 1)
	HGD + FT	EV	18.3	< 0.001 2)
		skn-1	18.3	
	HGD	*skn-1*	13.1	< 0.001 1)
	HGD + FT	*skn-1*	14.6	0.054 2)
2nd experiment		EV	19.9	
	HGD	EV	17.1	< 0.001 1)
	HGD + FT	EV	19.8	< 0.001 2
		skn-1	17.2	
	HGD	*skn-1*	15.4	< 0.001 1)
	HGD + FT	*skn-1*	15.6	0.360 2)
3rd experiment		EV	23.4	
	HGD	EV	19.4	< 0.001 1)
	HGD + FT	EV	23.8	< 0.001 2
		skn-1	20.1	
	HGD	*skn-1*	16.6	< 0.001 1)
	HGD + FT	*skn-1*	17.5	0.069 2)

1) *P* value was calculated using the log-rank test by comparing the survivals of no supplementation and HGD only with the same RNAi clone.

2) *P* value was calculated using the log-rank test by comparing the survivals of HGD only and HGD + FS with the same RNAi clone.

HGD, high glucose diet (40 mM glucose); FT, fisetin (0.1 g/L).

Table S4. Effect of fisetin on degeneration of dopaminergic neurons.

	Supplementation	Relative fluorescence (%)	*P* value
1st experiment		100.0 ± 3.27	
	6-OHDA	65.4 ± 2.96	< 0.001[1)]
	6-OHDA + L-DOPA	89.4 ± 4.91	< 0.001[2)]
	6-OHDA + FT	87.4 ± 3.36	< 0.001[2)]
2nd experiment		100.0 ± 2.63	
	6-OHDA	61.5 ± 4.12	< 0.001[1)]
	6-OHDA + L-DOPA	84.5 ± 4.17	< 0.001[2)]
	6-OHDA + FT	71.6 ± 3.36	0.021[2)]
3rd experiment		100.0 ± 2.17	
	6-OHDA	67.2 ± 2.64	< 0.001[1)]
	6-OHDA + L-DOPA	102.0 ± 3.66	< 0.001[2)]
	6-OHDA + FT	91.8 ± 3.27	< 0.001[2)]

[1)] *P* value was calculated using the Student's t test by comparing to untreated control.

[2)] *P* value was calculated using the Student's t test by comparing to 6-OHDA-treated group.

6-OHDA, 6-hydroxydopamine hydrobromide; L-DOPA, L-3,4-dihydroxyphenylalanine; FT, fisetin (0.1 g/L).

Table S5. Effect of *daf-16* or *bec-1* knockdown on lifespan extension by fisetin.

		Mean lifespan (d)		
	RNAi	Control	FT	*P* value[1)]
1st experiment	*daf-16*	12.3	12.0	0.746
	bec-1	18.8	17.5	0.155

2nd experiment	*daf-16*	10.2	10.2	0.714
	bec-1	18.1	18.7	0.913
3rd experiment	*daf-16*	11.4	11.3	0.392
	bec-1	17.2	15.0	0.064

1) *P* value was calculated using the log-rank test by comparing the survival of the untreated control group (0 g/L fisetin) to that of fisetin-treated group (0.1 g/L fisetin).

FT, fisetin (0.1 g/L).

Table S6. Primer set of each gene used for quantitative RT-PCR.

Gene Name	Primer	Sequence
ama-1	forward	5'-CGG AGC AGC CAG GAA CTT C-3'
	reverse	5'-AAC GGG AAA AAT CTT ATG AAT-3'
skn-1	forward	5'-CTC TCT TCT GGC ATC CTC TAC CA-3'
	reverse	5'-TTC TTG GAT TCT TCT TCT TGT TCG T-3'
ctl-1	forward	5'-AAT GGA TAC GGA GCG CAT AC-3'
	reverse	5'-GCG TCA GTT GGA TCG AGA TT-3'
sod-3	forward	5'-TGG TGG TGG ACA CAT CAA TC-3'
	reverse	5'-ACC GAA GTC GCG CTT AAT AG-3'
gst-4	forward	5'-GCT GAA GCC AAC GAC TCC AT-3'
	reverse	5'-GAC CGA ATT GTT CTC CAT CGA-3'
bec-1	forward	5'-AGG AGC TGG AGC AAC AGT TGA AGA-3'
	reverse	5'-ATA TTG ACG TTC GGC TTC CAG CGA-3'
lgg-1	forward	5'-AAC AAC TTT GAG AAG CGT CGT GCC-3'
	reverse	5'-TCT TCT GGA CGA AGT TGG ATG CGT-3'
